# Generalization across view in face memory and face matching

**DOI:** 10.1068/i0669

**Published:** 2014-11-24

**Authors:** Alejandro J. Estudillo, Markus Bindemann

**Affiliations:** School of Psychology, University of Kent, Canterbury, Kent, UK; e-mail: aje24@kent.ac.uk; School of Psychology, University of Kent, Canterbury, Kent, UK; e-mail: m.bindemann@kent.ac.uk

**Keywords:** view generalization, face recognition, face matching, individual differences, unfamiliar faces

## Abstract

While a change in view is considered to be one of the most damaging manipulations for facial identification, this phenomenon has been measured traditionally with tasks that confound perceptual processes with recognition memory. This study explored facial identification with a pairwise matching task to determine whether view generalization is possible when memory factors are minimised. Experiment 1 showed that the detrimental view effect in recognition memory is attenuated in face matching. Moreover, analysis of individual differences revealed that some observers can identify faces across view with perfect accuracy. This was replicated in Experiment 2, which also showed that view generalization is unaffected when only the internal facial features are shown. These results indicate that the view effect in recognition memory does not arise from data limits, whereby faces contain insufficient visual information to allow identification across views. Instead, these findings point to resource limits, within observers, that hamper such person identification in recognition memory.

## 1 Introduction

Changes in view can induce substantial variation in the appearance of a person's face. For example, while a frontal face displays a pair of eyes and a symmetrical mouth and nose, the same features are only partly visible in profile view. When we encounter familiar faces, of the people that we know, this variation presents little difficulty for person identification (see e.g. Eger, Schweinberger, Dolan, & Henson, 2005; Troje & Kersten, 1999). For unfamiliar faces, however, view changes appear to reduce identification accuracy dramatically.

A striking demonstration of this effect comes from recognition memory paradigms. In these studies, a set of unfamiliar faces is memorised in an initial learning phase. Recognition is then assessed at a subsequent test phase, in which the learned faces are intermixed with new identities. In an early study in this field, Bruce (1982) showed that observers recognise 90% of faces when these are presented at study and test in the same view. However, accuracy declined dramatically, to just 60%, when recognition memory was subsequently tested across a change in view. This effect has been replicated many times (see e.g. Hill, Schyns, & Akamatsu, 1997; Kaufmann, Schweinberger, & Burton, 2009; Krouse, 1981; O'Toole, Edelman, & Bülthoff, 1998) and appears to be sensitive to the degree of rotation between to-be-compared views. Thus, recognition memory declines linearly as the angle between study and test view increases (Longmore, Liu, & Young, 2008).

These findings suggest that the recognition of unfamiliar faces is highly viewpoint-dependent. A potential explanation for this effect is that the recognition of such unfamiliar faces across different views is a data-limited problem (Norman & Bobrow, 1975; see also Jenkins & Burton, 2011). Accordingly, one view of a face can provide only limited information about the appearance of the same person's face from a different view. As a consequence, recognition accuracy declines.

While accounts of such viewpoint-dependence have been considered in the face perception domain for some time (see e.g. Bruce & Young, 1986; Hill et al., 1997; Longmore et al., 2008; O'Toole et al., 1998), an alternative explanation is also possible. This is based on the idea that view-generalization is poor in recognition memory tasks because of resource limits, within observers. According to this explanation, faces might, in fact, contain sufficient visual information for the reliable identification across different views. However, observers cannot maximise the available visual information for this purpose in recognition memory tasks (for similar ideas, see Alenezi & Bindemann, 2013; Bindemann, Avetisyan, & Rakow, 2012; Liu & Chaudhuri, 2000).

One way of exploring this possibility is to compare person identification across different face views in a recognition memory paradigm with performance in a matching task. In matching paradigms, pairs of faces are presented simultaneously and observers have to decide whether these depict the same person or two different people (Johnston & Bindemann, 2013). This task has been used widely in theoretical (see e.g. Clutterbuck & Johnston, 2002; Hole, 1994; Megreya & Burton, 2006, 2007; Megreya, White, & Burton, 2011) and applied research on person identification (see e.g. Bindemann & Sandford, 2011; Kemp, Towell, & Pike, 1997). However, while matching performance correlates with recognition memory for faces (Burton, White, & McNeill, 2010; Megreya & Burton, 2006), matching tasks minimise the contribution of memory components in tests of face identification (Megreya & Burton, 2008). As a consequence, these tasks provide a more direct test for the contribution of perceptual components to facial identification than recognition memory paradigms.

With regard to view generalization, there is already some preliminary evidence that observers can match faces across the same and different views with near-similar levels of accuracy (Bindemann, Attard, Leach, & Johnston, 2013). This indicates that changes in view might be much less damaging for person identification than studies of recognition memory, which consistently report very large view effects, might suggest (see e.g. Bruce, 1982; Bruce et al., 1999; Hill et al., 1997; Longmore et al., 2008; O'Toole et al., 1998). Indeed, in Bindemann et al.'s (2013) study matching performance was worse across the same face view when image quality was degraded through pixelation, than across different face views when image quality was high. This indicates that view might exert a small effect on person identification in comparison to other factors.

So far, these findings are limited to a single experiment (Experiment 4 in Bindemann et al., 2013) and a direct comparison of view generalization in face memory and face matching has not been made. The aim of the present research is therefore to provide such a comparison. In Experiment 1, observers performed both a matching task and a recognition memory task for faces shown in the same view (frontal, frontal) and in two different views (frontal, profile). Experiment 2 then explored the effect of view on face matching further, by comparing performance with full faces with a condition in which only the internal facial features were shown. The main aim of these experiments was to determine the extent to which person identification across views is possible when memory factors are minimised. In turn, this should reveal whether the perceptual information that is present in frontal and profile faces is sufficient for view generalization.

## 2 Experiment 1

In this experiment, observers performed both a recognition memory and a matching task with faces. In the recognition task, observers first studied a set of faces in a frontal view. This was followed by a test phase, in which these faces were intermixed with previously unseen faces. At test, faces were shown either in a frontal or a profile view. In the matching task, on the other hand, pairs of faces were shown simultaneously and required identity match (i.e. both faces depict the same person) or mismatch decisions (two different people are shown). In a pair, both faces were either shown in the same view (two frontal faces) or different views (a frontal and a profile face).

We expected performance to be generally worse in recognition memory than face matching, due to the added memory demands. In addition, we also expected to find the consistent effect of face view that has been reported with recognition memory paradigms (e.g. Hill et al., 1997; Kaufmann et al., 2009; Longmore et al., 2008). The question of main interest concerned the extent to which view generalization would be possible in the matching task. If view generalization reflects a data-limited problem, whereby faces do not provide sufficient perceptual information to allow for person identification across different views, then accuracy for both face memory and face matching should decline under these conditions. If, on the contrary, this is a resource-limited problem, whereby observers cannot perform this task from memory, then matching performance should be more comparable for face pairs comprising images of the same or different views.

To fully address this question, we analyzed the data by comparing mean accuracy, across a group of observers, for the view conditions. In addition, we also assessed individual differences in accuracy, between observers, within these tasks. This analysis was included to determine whether some individuals can consistently identify faces across different views even when a group of observers cannot. Such a result would also suggest that the problem of view generalization is, in principle, solvable (for similar approaches, see e.g. Bindemann et al., 2012).

### 2.1 Method

#### 2.1.1 Participants

Forty undergraduate students (34 females) from the University of Kent, with a mean age of 20 years (SD = 3.7), participated in the experiment for course credits. All reported normal or corrected-to-normal vision. This experiment was conducted in accordance with the Helsinki Declaration (2008).

#### 2.1.2 Stimuli

One hundred and sixty pairs of male and female faces were taken from the Glasgow Face Matching Test (Burton et al., 2010) for the matching task (80 pairs) and the recognition memory task (80 pairs). The allocation of faces to these tasks was counterbalanced across observers. Thus, the same face identities were not encountered by the same observers in the memory and the matching task. However, over the course of the experiment, the 160 face pairs were rotated across observers, so that they were seen equally often in each task.

**Figure 1.**
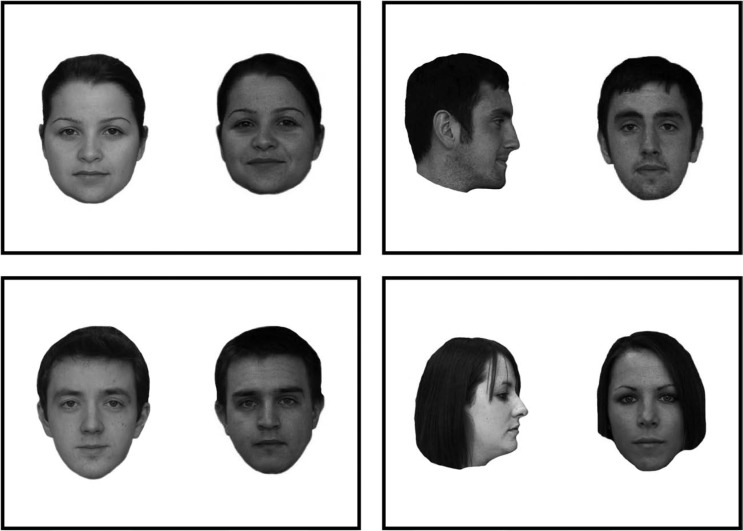
Example face pairs, depicting identity matches (top) and mismatches (bottom) in the same (left) and different views (right).

All faces were shown in greyscale on a white background and measured maximally 350 pixels in width at a resolution of 72 ppi. Half of these pairs depicted two frontal views (same-view condition), while the other half depicted one face in frontal and one face in a profile view (different-view condition). In addition, half of these pairs comprised identity matches, in which two different photographs of the same person were shown, whereas the other half depicted mismatches, which showed the faces of two different identities. Finally, one face photograph in each pair was taken with a high-quality digital camera, while the other was a still frame from high-quality video. This was done to ensure that, even across the same face view, the resulting images provide similar but not identical images of a person. This ensures that the task cannot be performed using simple pictorial matching/memory (see e.g. Burton et al., 2010; Jenkins & Burton, 2011). Example stimuli are shown in Figure 1.

#### 2.1.3 Procedure

The experiment was run on E-Prime software and comprised the recognition memory and the matching task. The order of these tasks was counterbalanced across observers. Accuracy was emphasised in both.

Recognition memory task: This task consisted of an initial encoding stage and a subsequent recognition stage. Each trial began with a central fixation cross, which was shown for one second. This was followed by a single face in the centre of the screen. Observers were asked to learn these faces for a subsequent recognition test. Learning was self-paced, so observers pressed a key to move on to the next face when they felt ready to do so. Each observer completed 40 learning trials, comprising 40 different facial identities.

Following the learning phase, observers were given a short filler task that involved simple number comparisons and took approximately two minutes to complete. This was followed by the recognition test. In this task, the learned faces were presented in a randomly intermixed order with 40 new faces, which had not been seen previously in the experiment. Each trial began with a one-second fixation cross. This was followed by a face, which required old (i.e. previously seen in the learning phase) or new (previously unseen) decisions by pressing one of two buttons on a response box. Old and new test faces were equally likely to appear in a frontal or profile view. There was no time limit for performing the task.

Matching task: This task consisted of 40 match and 40 mismatch trials, which were shown in a random order. In the match and mismatch trials, both faces in a pair were equally likely to be shown in the same view (i.e. two frontal faces) or a different view (a frontal and a profile face). On each trial, observers were shown a central fixation cross for one second. This was replaced by a face pair stimulus display, which remained onscreen until a response was registered. Observers were asked to classify the face pairs as identity matches or mismatches by pressing one of two buttons on a response box. Once again, there was no time limit for this task.

### 2.2 Results

#### 2.2.1 Recognition memory

**Figure 2.**
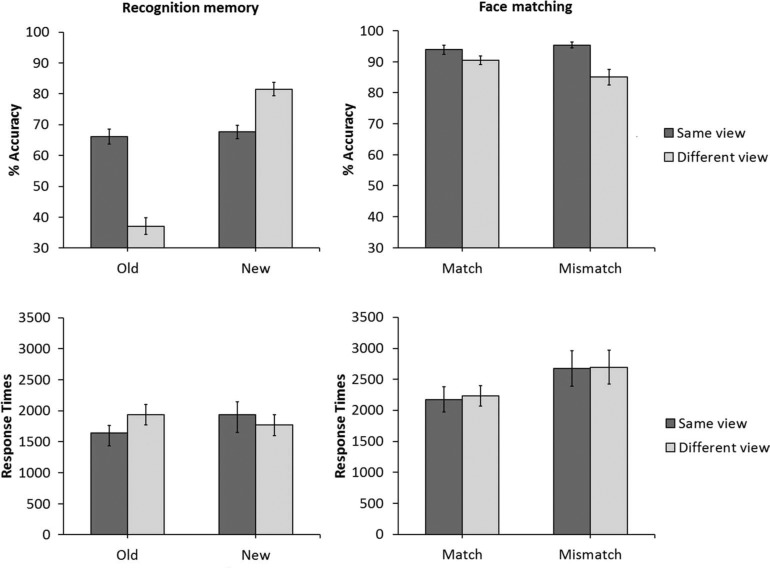
The percentage of correct responses (top) and response times (bottom) for the recognition memory task and the matching task in Experiment 1. Error bars denote standard error of the mean.

Performance for the recognition memory task was analysed first. Figure 2 shows the mean percentage of correct old and new responses for the same- and different-view conditions. Across the same view, observers correctly recognised 66% of faces from the learning phase, but accuracy dropped to just 37% when recognition was tested with a different face view. This pattern was also reflected in the percentage of new responses, as observers were more likely to classify different-view faces as previously unseen than same-view faces. These observations were confirmed by a 2 (trial type: old versus new) × 2 (view: same- versus different-view) within-subject ANOVA, which showed a main effect of trial type, *F*(1,39) = 52.57, *p* < .01, η^2^_p_ = .57, a main effect of view, *F*(1,39) = 21.92, *p* < .01, η^2^_p_ = .31, and an interaction between both factors, *F*(1,39) = 85.87, *p* < .01, η^2^_p_ = .68. Analysis of simple main effects showed an effect of view for old trials, *F*(1,39) = 106.14, *p* < .01, η^2^_p_ = .73, which reflects higher recognition accuracy across the same view than across different views. A simple main effect of view also arose for new trials, *F*(1,39) = 24.07, *p* < .01, η^2^_p_ = .38, as more new responses were made in the different- than the same-view condition. In addition, a simple main effect of trial type was also observed in the different-view condition, *F*(1,39) = 109.28, *p* < .01, η^2^_p_ = .73, due to the higher percentage of new as compared to old responses. The simple main effect of trial type for the same-view condition was not significant, *F*(1,39) = 0.17, *p* = .67, η^2^_p_ = .01.

Although task instructions emphasised accuracy, response times were analysed also for completeness. The cross-subject means of the median response times for all correct trials are shown in Figure 2. A 2 (trial type: old versus new) × 2 (view: same- versus different-view) within-subject ANOVA of this data did not show a main effect of trial type, *F*(1,39) = 0.51, *p* = .47, η^2^_p_ = .01, or view, *F*(1,39) = 0.69, *p* = .41, η^2^_p_ = .18, but an interaction between both factors was found, *F*(1,39) = 6.37, *p* < .05, η^2^_p_ = .14. Analysis of simple main effects showed an effect of view for old trials, *F*(1,39) = 5.27, *p* < .05, η^2^_p_ = .12, which reflects slower responses for different- than same-view trials, but not for new trials, *F*(1,39) = 2.35, *p* = .13, η^2^_p_ = .05. A simple main effect of trial type was also observed in the same-view condition, *F*(1,39) = 4.17, *p* < .05, η^2^_p_ = .09, with slower responses to new than to old faces. The corresponding simple main effect was not significant for the different-view condition, *F*(1,39) = 2.29, *p* = .13, η^2^_p_ = .05.

#### 2.2.2 Matching task

The percentage of correct responses for the matching task are also shown in Figure 2. These data show that matching performance was also lower in the different-view than in the same-view condition, both on identity match and mismatch trials. However, compared to the recognition memory task, accuracy was generally higher and the difference between view condition was much reduced, at 3% on match trials and 10% on mismatch trials. A 2 (trial type: match versus mismatch) × 2 (view: same- versus different-view) within-subject ANOVA did not find a main effect of trial type, *F*(1,39) = 0.93, *p* = .34, η^2^_p_ = .02. However, a main effect of view was found, *F*(1,39) = 26.84, *p* < .01, η^2^_p_ = .41, which reflects a reduction in matching accuracy in the different-view condition compared to same-view trials. The interaction between trial type and view also approached significance, *F*(1,39) = 4.06, *p* = .051, η^2^_p_ = .09. For this reason, this interaction is also explored further here. Analysis of simple main effects did not show an effect of view for match trials, *F*(1,39) = 3.29, *p* = .08, η^2^_p_ = .07. However, a simple main effect of view was found for mismatch trials, *F*(1,39) = 17.64, *p* < .01, η^2^_p_ = .31, as accuracy was higher in the same-view than the different-view condition.

The median correct response times were analysed again for completeness (see Figure 2). A 2 (trial type: match versus mismatch) × 2 (view: same- versus different-view) within-subject ANOVA of this data showed a main effect of trial type, *F*(1,39) = 8.01, *p* < .01, η^2^_p_ = .17, due to faster responses on match than on mismatch trials. The main effect of view, *F*(1,39) = 0.10, *p* = .74, η^2^_p_ = .01, and the interaction between trial type and view were not significant, *F*(1,39) = 0.17, *p* = .89, η^2^_p_ = .01.

#### 2.2.3 Individual differences

**Figure 3.**
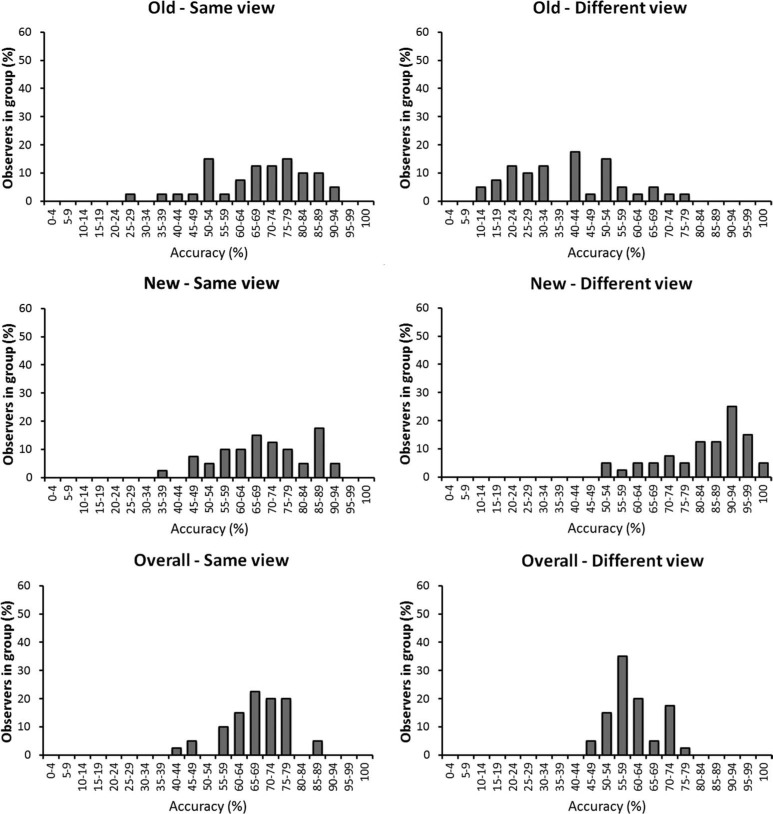
Individual differences in the recognition memory task in Experiment 1, grouped by the accuracy that observers achieved.

Mean accuracy in the matching task was relatively high. Considering that this measure summarises performance for a group of forty participants, it is plausible that some individuals performed this task with perfect accuracy. To explore this possibility, we grouped observers according to their accuracy. For the recognition memory task, data are provided in Figure 3 and show that none of the observers could recognise faces (as old) across the same face view with 95% accuracy or more. However, this threshold was even lower, at 80%, for recognition across different views. Indeed, very few participants (17%) achieved an accuracy level of 55% or over in this condition.

**Figure 4.**
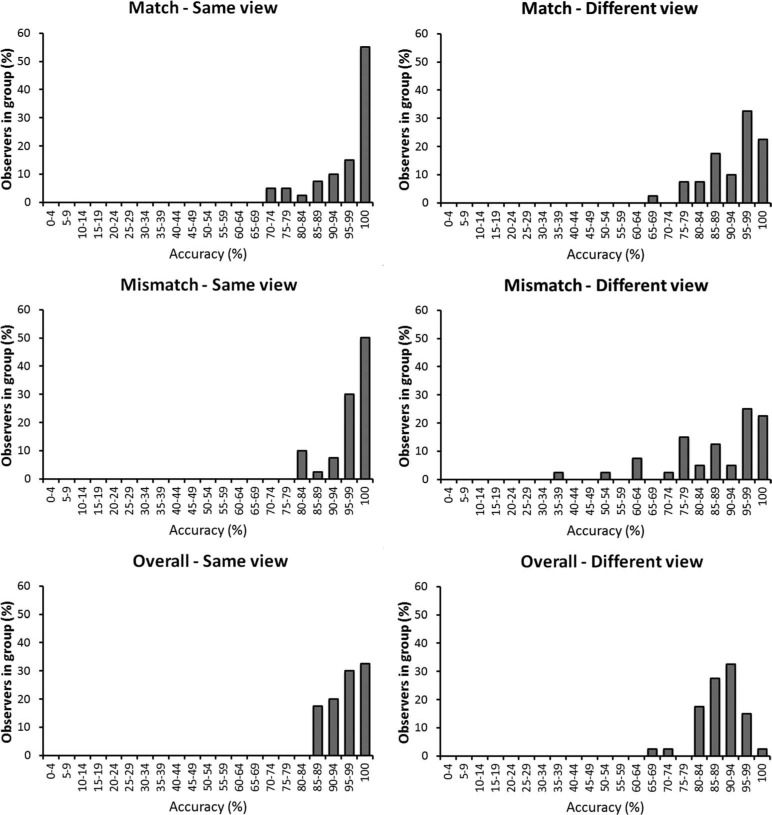
Individual differences in the face matching task in Experiment 1, grouped by the accuracy that observers achieved.

Accuracy was generally higher in the matching than the memory task (see Figure 4). This contrast is particularly striking when individual differences in performance are considered. For example, whereas 95% of observers could match faces across different views with at least 80% accuracy, none reached such accuracy in the recognition memory task. Moreover, a subset of observers (18%) matched faces in the different-view condition with near-perfect or perfect accuracy (95–100%).

### 2.3 Discussion

This experiment compared identification performance in a recognition and a matching task to examine whether generalisation across different face views is possible when memory factors are minimised. Performance in the recognition memory task was error-prone across the same face view and was poorer still in the different-view condition. Moreover, the pattern of results suggests a response bias, whereby observers were also more likely to classify faces as new when these were encountered in a different test view. These findings converge with previous studies that have shown limited generalisation across views in recognition memory paradigms (e.g. Hill et al., 1997; Kaufmann et al., 2009; Krouse, 1981; Longmore et al., 2008; O'Toole et al., 1998). In the current experiment, this view effect is perhaps particularly striking considering the accuracy of individual observers. For example, whereas at least some observers recognised faces with 90–94% accuracy across the same view, none achieved more than 79% accuracy across different face views, and the majority of observers were correct on less than 50% of trials in this condition (see Figure 3). On its own, these data therefore support the notion that view generalisation for unfamiliar faces is rather limited.

A different picture emerged on the matching task. In this case, accuracy was generally higher. Indeed, while a view effect was still found, the mean accuracy for the different-view condition was at over 87%. This indicates that, more often than not, generalisation across views is possible in facial identification when memory factors are minimised. This contrast with the recognition task is particularly striking considering the performance of individual observers. For example, whereas the majority of observers (95%) achieved an overall matching accuracy of at least 80% across different face views, none reached this level of accuracy in the recognition memory task. Moreover, a subset of observers could match different views of faces with near-perfect (95–99%) or perfect (100%) accuracy. This is an important finding because it demonstrates that this task is, in fact, solvable. This indicates that the problem of view generalisation in face memory, and to a lesser extent in face matching, is not caused by data limits, whereby faces contain insufficient visual information to make identification across views possible. Instead, these findings point to a resource limit, whereby observers cannot perform this task well from memory.

## 3 Experiment 2

Experiment 1 indicates that generalisation across views is possible in facial identification when memory factors are minimised. Before reaching this strong conclusion, we sought to replicate these results with a further experiment. In addition, Experiment 2 also contrasted matching of the entire face with a condition in which the internal facial features were preserved (i.e. the eyes, nose, mouth) but external features, such as hairstyle, were removed. Such external features provide a salient context that can improve recognition and matching performance (see, e.g. Bruce et al., 1999; Ellis, Shepherd, & Davies, 1979; Endo, Takahashi, & Maruyama, 1984). However, these changeable features can also provide misleading identity information (see, e.g. Frowd et al., 2012; Sinha & Poggio, 1996, 2002) and dominate the identification of unfamiliar faces (Bonner, Burton, & Bruce, 2003; Clutterbuck & Johnston, 2005; Young, Hay, McWeeney, Flude, & Ellis, 1985). This raises the possibility that the results of the matching task in Experiment 1 do not reflect generalisation across different views of faces per se, but are driven by the external features of these stimuli. The removal of these features in Experiment 2 should therefore focus the task on the most relevant facial identity information.

## 3.1 Method

### 3.1.1 Participants

Twenty undergraduate students (13 females) from the University of Kent, with a mean age of 20 years (SD = 2.1), participated for course credits or a small payment. All reported normal or corrected-to-normal vision. This experiment was conducted in accordance with the Helsinki Declaration (2008).

### 3.1.2 Stimuli and procedure

The stimuli and procedure were identical to Experiment 1, except for the following changes. The current experiment only comprised the matching task, but the face stimuli were now presented with external features intact (40 match trials and 40 mismatch trials) or with the external features removed (also 40 match and 40 mismatch trials). This was achieved by cropping the faces to an elliptical shape that revealed only the area of the internal facial features (i.e. the eyes, nose, mouth).

In the experiment, these conditions were presented in a randomly intermixed order, for a total of 160 trials per participant. However, over the course of the experiment, the presentation of the face stimuli was counterbalanced across observers so that each face pair was encountered in the full-face and internal-feature condition an equal number of times. Observers were asked to classify these stimuli as identity matches or mismatches, regardless of whether the faces in a pair were shown in the same or different views.

### 3.2 Results

#### 3.2.1 Overall matching accuracy

**Figure 5.**
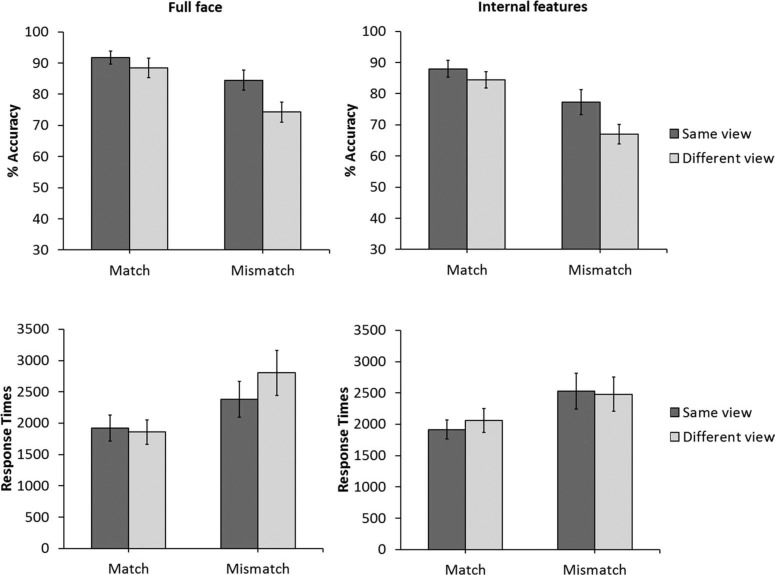
The percentage of correct responses (top) and response times (bottom) for the full-face and the internal feature conditions in Experiment 2. Error bars denote standard error of the mean.

The percentage of correct responses is shown in Figure 5 as a function of experimental condition. A 2 (face: full-face versus internal-features) × 2 (view: same versus different view) × 2 (trial type: match versus mismatch) within-subject ANOVA of these data showed a main effect of trial type, *F*(1,19) = 13.54, *p* < .01, η^2^_p_ = .41, due to higher accuracy on identity match than mismatch trials. A main effect of view was also found, *F*(1,19) = 23.37, *p* < .01, η^2^_p_ = .55, which reflects better matching performance for same-view than different-view face pairs. In addition, the main effect of face was significant, *F*(1,19) = 19.75, *p* < .01, η^2^_p_ = .51, as accuracy was higher in the full-face than the internal-feature conditions. None of the interactions were significant, all *F'*s ≤ 1.69, *p'*s ≥ .21.

The median correct response times were also analysed for completeness (see Figure 5). A 2 (face: full-face versus internal-features) × 2 (view: same versus different view) × 2 (trial type: match versus mismatch) within-subject ANOVA of these data showed a main effect of trial type, *F*(1,19) = 13.92, *p* < .01, η^2^_p_ = .40, due to slower responses on mismatch trials. None of the other main effects or interactions were significant, all *F'*s ≤ 3.00, *p'*s ≥ .10.

#### 3.2.2 Individual differences

**Figure 6.**
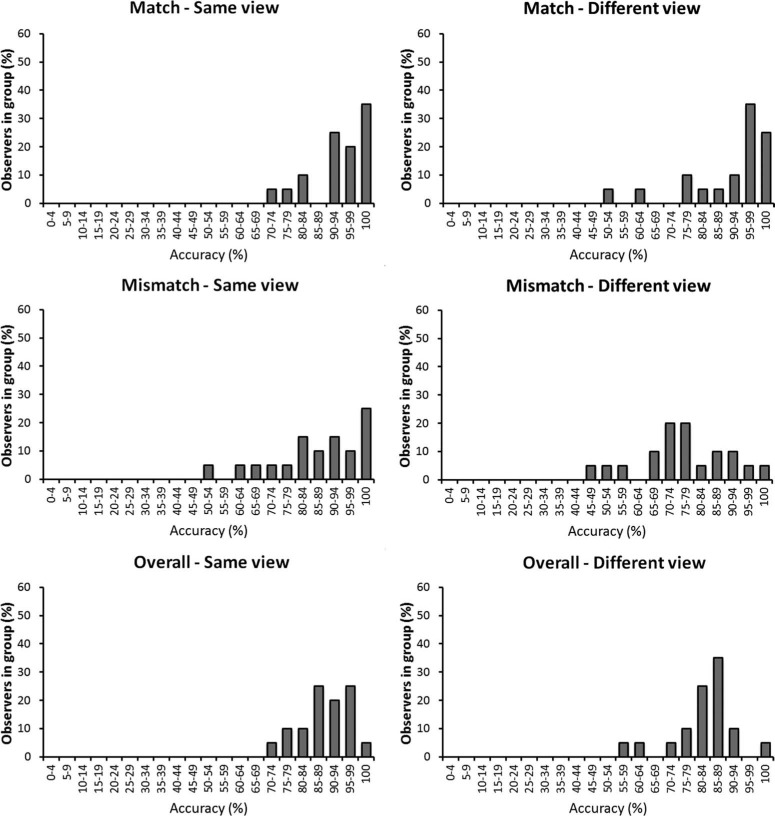
Individual differences in the full-face condition of Experiment 2, grouped by the accuracy that observers achieved.

To explore whether mean performance reflects data or resource-limits, we again turn to an inspection of the individual observer data. As in Experiment 1, these data show that many observers can perform this task with very high accuracy in the full-face condition when both faces in a pair are shown in the same view (see Figure 6). Moreover, even across different face views, about 15% of observers can still perform this task with perfect (100%) or near-perfect (90–94%) accuracy.

**Figure 7.**
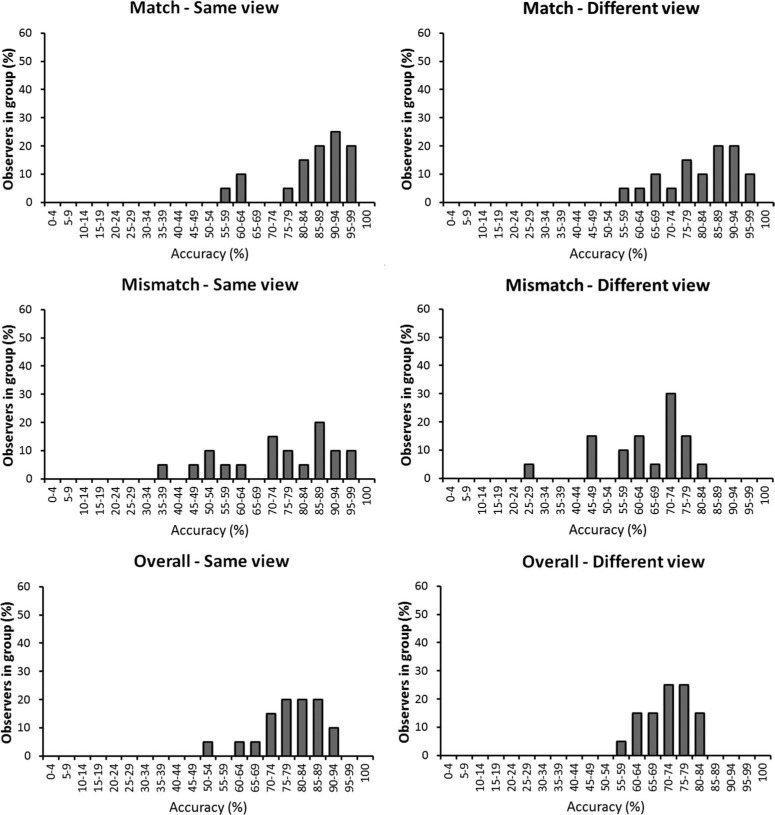
Individual differences in the internal feature condition of Experiment 2, grouped by the accuracy that observers achieved.

In line with the mean accuracy data, individual performance was also lower in the internal feature conditions, where none of the observers achieved 100% accuracy (see Figure 7). However, some 70% and 40% of observers still achieved an overall accuracy level of at least 75% in the same- and different-view conditions, respectively.

### 3.3 Discussion

This experiment replicates the results of the matching task in Experiment 1. Accuracy was higher during the comparison of faces in the same view than across different views. As in Experiment 1, however, this effect was numerically small and a subset of participants was able to match faces with perfect accuracy regardless of variation in view. In addition, accuracy for full-face displays was compared with internal feature displays to determine whether view generalisation remains possible when salient external facial features are eliminated. In line with previous research, matching performance was better when the entire face was shown than when only the internal facial features were preserved (see, e.g. Bonner et al., 2003; Ellis et al., 1979). Importantly, however, this effect did not interact with view. This indicates that the removal of external features impairs facial identification generally, independent of any view effects.

## 4 General discussion

A change in view is considered to be one of the most detrimental manipulations for the recognition of unfamiliar faces (see, e.g. Johnston & Edmonds, 2009). A possible explanation for this effect is that one view of a face can only provide limited information about the same person's face from a different view. As a result of such perceptual data limits, identification accuracy might decline. In this study, we investigated an alternative explanation, which is based on the notion that viewpoint-dependence might reflect internal resource limits, within observers. According to this view, faces might, in fact, contain sufficient information to make identification possible even across drastic changes in view (e.g. from frontal to profile). However, observers might not be able to utilise this information fully in recognition memory tasks.

To explore this possibility, we compared recognition memory for faces with a matching task. In line with previous studies, generalisation across views was poor from memory (e.g. Hill et al., 1997; Krouse, 1981; Kaufmann et al., 2009; Longmore et al., 2008; O'Toole et al., 1998). This effect was such that most observers recognised at least half of all faces when these were shown in the same view at learning and test, but recognised less than half of the faces across different views. On its own, these data therefore support the notion that view generalisation in the identification of unfamiliar faces is rather limited. A different picture emerged, however, on the matching task. While matching accuracy was also lower across different face views than across the same view, most observers (95%) achieved an overall cross-view matching accuracy of at least 80% in Experiment 1. Moreover, a subset of observers could match faces with perfect (100%) accuracy across different views. This finding was replicated in Experiment 2, which also showed that some observers can still identify faces across views with relatively high accuracy, of about 80%, from the internal features alone. These findings are important for demonstrating that faces can be identified consistently across different views. Moreover, the contrast with the recognition memory task suggests that the problem of view generalisation is not caused by data limits, whereby faces contain insufficient visual information to make identification across views possible. Instead, these findings point to a resource limit, whereby observers do not have the capacity to perform this task well from memory.

We draw these conclusions with some caveats. Considering that accuracy was higher in face matching than recognition memory, the possibility arises that the impact of a change in view on the former task might have been masked by ceiling effects. In line with this reasoning, view effects appear to be more pronounced in face matching under more taxing conditions, in which a target has to be compared with multiple identities (see e.g. Benton, Sivan, Hamsher, Varney, & Spreen, 1983; Bruce et al., 1999). However, it is also noteworthy that recognition memory for faces in Experiment 1, despite utilising the same stimuli as the matching task, was poor. For example, mean recognition accuracy of previously seen faces dropped to just 37% when this was tested with a different view, and some individuals recognised as few as 10% of these faces. This indicates that it might be difficult to reduce general performance in the matching task without creating a concurrent floor effect in recognition memory.

It is also conceivable that general increases in task difficulty will not directly affect view generalisation in face matching. Whereas the current study employed optimised face photographs that provide a measure of best-possible performance (see Burton et al., 2010), matching accuracy can be reduced, for example, if to-be-compared face photographs vary in terms of image quality (Bindemann et al., 2013), lighting (Liu, Chen, Han, & Shan, 2013), expression (Jenkins, White, Van Montfort, & Burton, 2011) or age (Megreya, Sandford, & Burton, 2013). However, these additional factors also increase the difficulty of face matching across the same face view (akin to the internal feature manipulation in Experiment 2). This indicates that image-dependence, which reflects the similarity of two face photographs on a number of dimensions (e.g. image-quality, expression, lighting), and view-dependence are dissociable in face matching.

In summary, the current experiments suggest that different photographs of faces share sufficient perceptual information to support person identification even across drastically different views (e.g. from frontal to profile). In turn, the current data indicate that many identification errors across, and within, face views arise from internal processing limits, within observers (for similar suggestions, see Bindemann et al., 2012; Megreya & Bindemann, 2013). This distinction has been neglected in research on view generalisation in the face domain.

## References

[R1] Alenezi H. M., Bindemann M. (2013). The effect of feedback on face-matching accuracy. Applied Cognitive Psychology.

[R2] Benton A. L., Sivan A. B., Hamsher K., Varney D. S., Spreen N. R. O. (1983). Facial recognition: Stimulus and multiple choice pictures.

[R3] Bindemann M., Attard J., Leach A., Johnston R. A. (2013). The effect of image pixelation on unfamiliar-face matching. Applied Cognitive Psychology.

[R4] Bindemann M., Avetisyan M., Rakow T. (2012). Who can recognize unfamiliar faces? Individual differences and observer consistency in person identification. Journal of Experimental Psychology: Applied.

[R5] Bindemann M., Sandford A. (2011). Me, myself, and I: Different recognition rates for three photo-IDs of the same person. Perception.

[R6] Bonner L., Burton A. M., Bruce V. (2003). Getting to know you: How we learn new faces. Visual Cognition.

[R7] Bruce V. (1982). Changing faces: Visual and non-visual coding processes in face recognition. British Journal of Psychology.

[R8] Bruce V., Henderson Z., Greenwood K., Hancock P. J. B., Burton A. M., Miller P. (1999). Verification of face identities from images captured on video. Journal of Experimental Psychology: Applied.

[R9] Bruce V., Young A. (1986). Understanding face recognition. British Journal of Psychology.

[R10] Burton A. M., White D., McNeill A. (2010). The Glasgow Face Matching Test. Behavior Research Methods.

[R11] Clutterbuck R., Johnston R. A. (2002). Exploring levels of face familiarity by using an indirect face-matching measure. Perception.

[R12] Clutterbuck R., Johnston R. A. (2005). Demonstrating how unfamiliar faces become familiar using a face matching task. European Journal of Cognitive Psychology.

[R13] Eger E., Schweinberger S. R., Dolan R. J., Henson R. N. (2005). Familiarity enhances invariance of face representations in human ventral visual cortex: fMRI evidence. Neuroimage.

[R14] Ellis H. D., Shepherd J. W., Davies G. M. (1979). Identification of familiar and unfamiliar faces from internal and external features: Some implications for theories of face recognition. Perception.

[R15] Endo M., Takahashi K., Maruyama K. (1984). Effects of observer's attitude on the familiarity of faces: Using the difference in cue value between central and peripheral facial elements as an index of familiarity. Tohoku Psychologica Folia.

[R16] Frowd C. D., Skelton F., Atherton C., Pitchford M., Hepton G., Holden L., Hancock P. J. B. (2012). Recovering faces from memory: The distracting influence of external facial features. Journal of Experimental Psychology: Applied.

[R17] Hill H., Schyns P. G., Akamatsu S. (1997). Information and viewpoint dependence in face recognition. Cognition.

[R18] Hole G. J. (1994). Configurational factors in the perception of unfamiliar faces. Perception.

[R19] Jenkins R., Burton A. M. (2011). Stable face representations. Philosophical Transactions of the Royal Society B: Biological Sciences.

[R20] Johnston R. A., Bindemann M. (2013). Introduction to forensic face matching. Applied Cognitive Psychology.

[R21] Johnston R. A., Edmonds A. J. (2009). Familiar and unfamiliar face recognition: A review. Memory.

[R22] Kaufmann J. M., Schweinberger S. R., Burton A. M. (2009). N250 ERP correlates of the acquisition of face representations across different images. Journal of Cognitive Neuroscience.

[R23] Kemp R., Towell N., Pike G. (1997). When seeing should not be believing: Photographs, credit cards and fraud. Applied Cognitive Psychology.

[R24] Krouse F. L. (1981). Effects of pose, pose change, and delay on face recognition performance. Journal of Applied Psychology.

[R25] Liu C. H., Chaudhuri A. (2000). Recognition of unfamiliar faces: Three kinds of effects. Trends in Cognitive Sciences.

[R26] Liu C. H., Chen W., Han H., Shan S. (2013). Effects of image preprocessing on face matching and recognition in human observers. Applied Cognitive Psychology.

[R27] Longmore C. A., Liu C. H., Young A. W. (2008). Learning faces from photographs. Journal of Experimental Psychology: Human Perception and Performance.

[R28] Megreya A. M., Bindemann M. (2013). Individual differences in personality and face identification. Journal of Cognitive Psychology.

[R29] Megreya A. M., Burton A. M. (2006). Unfamiliar faces are not faces: Evidence from a matching task. Memory and Cognition.

[R30] Megreya A. M., Burton A. M. (2007). Hits and false positives in face matching: A familiarity based dissociation. Perception and Psychophysics.

[R31] Megreya A. M., Burton A. M. (2008). Matching faces to photographs: Poor performance in eyewitness memory (without the memory). Journal of Experimental Psychology: Applied.

[R32] Megreya A. M., Sandford A., Burton A. M. (2013). Matching face images taken on the same day or months apart: The limitations of photo ID. Applied Cognitive Psychology.

[R33] Megreya A. M., White D., Burton A. M. (2011). The other-race effect does not rely on memory: Evidence from a matching task. Quarterly Journal of Experimental Psychology.

[R34] Norman D. A., Bobrow D. G. (1975). On data-limited and resource-limited processes. Cognitive Psychology.

[R35] O'Toole A. J., Edelman S., Bülthoff H. H. (1998). Stimulus-specific effects in face recognition over changes in viewpoint. Vision Research.

[R36] Sinha P., Poggio T. (1996). I think I know that face. Nature.

[R37] Sinha P., Poggio T. (2002). United we stand: The role of head-structure in face recognition. Perception.

[R38] Troje N. F., Kersten D. (1999). Viewpoint-dependent recognition of familiar faces. Perception.

[R39] Young A. W., Hay D. C., McWeeny K. H., Flude B. M., Ellis A. W. (1985). Matching familiar and unfamiliar faces on internal and external features. Perception.

